# Aeration mitigates endoplasmic reticulum stress in *Saccharomyces cerevisiae* even without mitochondrial respiration

**DOI:** 10.15698/mic2021.04.746

**Published:** 2021-03-31

**Authors:** Huong Thi Phuong, Yuki Ishiwata-Kimata, Yuki Nishi, Norie Oguchi, Hiroshi Takagi, Yukio Kimata

**Affiliations:** 1 Graduate School of Science and Technology, Nara Institute of Science and Technology, 8916-5 Takayama, Ikoma, Nara, 630-0192, Japan.

**Keywords:** mitochondria, yeast, respiration, endoplasmic reticulum, stress, unfolded protein response

## Abstract

*Saccharomyces cerevisiae* is a facultative anaerobic organism that grows well under both aerobic and hypoxic conditions in media containing abundant fermentable nutrients such as glucose. In order to deeply understand the physiological dependence of *S. cerevisiae* on aeration, we checked endoplasmic reticulum (ER)-stress status by monitoring the splicing of *HAC1* mRNA, which is promoted by the ER stress-sensor protein, Ire1. *HAC1*-mRNA splicing that was caused by conventional ER-stressing agents, including low concentrations of dithiothreitol (DTT), was more potent in hypoxic cultures than in aerated cultures. Moreover, growth retardation was observed by adding low-dose DTT into hypoxic cultures of *ire1*Δ cells. Unexpectedly, aeration mitigated ER stress and DTT-induced impairment of ER oxidative protein folding even when mitochondrial respiration was halted by the ρ^o^ mutation. An ER-located protein Ero1 is known to directly consume molecular oxygen to initiate the ER protein oxidation cascade, which promotes oxidative protein folding of ER client proteins. Our further study using *ero1*-mutant strains suggested that, in addition to mitochondrial respiration, this Ero1-medaited reaction contributes to mitigation of ER stress by molecular oxygen. Taken together, here we demonstrate a scenario in which aeration acts beneficially on *S. cerevisiae* cells even under fermentative conditions.

## INTRODUCTION

The endoplasmic reticulum (ER) is a flat or tubular-shaped membranous sac, which facilitates the folding and maturation of proteins that are subsequently transported to the cell surface or other organelles. Protein folding in the ER frequently accompanies formation of the disulfide bond between two cysteine residues, and is therefore called oxidative protein folding [1]. While protein disulfide-isomerases (PDIs) directly promote the oxidative folding of ER client proteins, Ero1 mediates the oxidation of PDI [1, 2]. In other words, Ero1 initiates an ER oxidation cascade that contributes to the disulfide-bond formation and oxidative folding of ER client proteins. Moreover, proteins are subjected to the N-linked glycosylation in the ER.

Dysfunction of the ER is tightly linked to accumulation of unfolded proteins in the ER, and is known as ER stress. Eukaryotic cells commonly alter their transcriptome profile in response to ER stress. This cellular protective response is called the unfolded protein response (UPR), and is mediated, at least in part, by the ER-located transmembrane protein Ire1, which works as an endoribonuclease [3]. In the budding yeast *Saccharomyces cerevisiae*, Ire1 is activated strictly in an ER stress-dependent manner, and facilitates the splicing of *HAC1* mRNA, which is then translated into a transcription-factor protein that is responsible for the UPR [4].

Unlike many other widely known eukaryotic species, *S. cerevisiae* is a facultative anaerobic organism. In the presence of abundant fermentable sugars, such as glucose, *S. cerevisiae* quickly produces ATP via glycolysis and performs ethanol fermentation [5]. *S. cerevisiae* cells are usually grown under aerobic conditions with shaking in many laboratories for basic-biology researches, whereas industrial ethanol fermentation is frequently performed under more hypoxic conditions in batch fermentation tanks. In order to bridge the knowledge from these two different growth conditions, it is important to understand how aerobic agitation affects the physiological status of *S. cerevisiae*.

In this context, we compared the cellular response of *S. cerevisiae* cells to ER-stressing stimuli by culturing with and without aerobic agitation, and observed the followings: (1) ER stress is not induced by hypoxic conditions alone; (2) meanwhile, ER stress induced by conventional stress stimuli is aggravated when cells are cultured without aeration; (3) this phenomenon is observed even when mitochondrial respiration is halted. We propose that molecular oxygen mitigates ER stress via both mitochondrial respiration and Ero1-mediated oxidative protein folding.

## RESULTS

**Figure 1:**
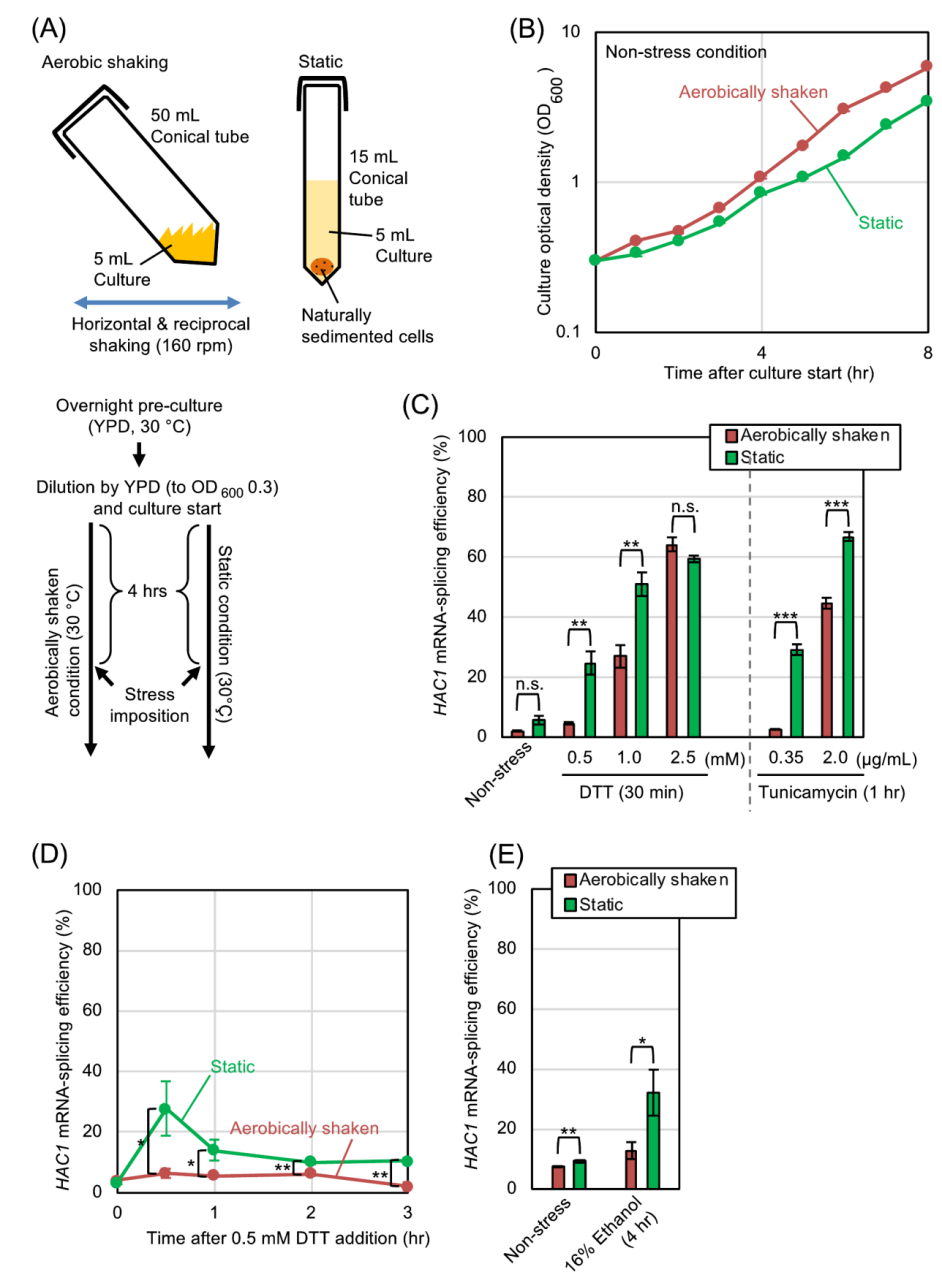
FIGURE 1: *HAC1*-mRNA splicing is compromised by aerobic agitation. **(A)** Procedures for culturing and ER-stress induction of *S. cerevisiae* cells under the aerobically shaken condition or under the static condition. ER-stress agents (DTT, tunicamycin, or ethanol) were added to the media 4-hr after culture start. **(B)** Optical density of cultures (wild-type BY4742 cells) was monitored under non-stressing conditions. **(C-E)** Wild-type BY4742 cells were cultured and were ER-stressed (or remained non-stressed) as shown in panel A, and were checked for *HAC1*-mRNA splicing. n.s. (not significant): p > 0.05, *: p < 0.05, **: p < 0.01, ***: p < 0.001.

At the beginning of this study, we examined how aerobic shaking of cultures affects the splicing of *HAC1* mRNA, which represents ER-stressing status, in *S. cerevisiae* cells. The protocol that was used for culturing is shown in **Fig. 1A**. After pre-culturing overnight under aerobic conditions, we further cultured *S. cerevisiae* cells either under the aerobically shaken or static conditions. **Fig. 1B** shows that the growth of *S. cerevisiae* cells was slightly slower under the static condition. Throughout this study, the splicing of *HAC1* mRNA was monitored by RT-PCR analysis of total RNA samples, and the splicing efficiency was quantitatively measured as described in our previous study [6]. The *HAC1*-mRNA splicing was almost negligible in the absence of ER-stress stimuli under both the aerobically shaken condition and the static condition, indicating that aeration alone does not change the ER-stressing status in *S. cerevisiae* (**Fig. 1C** (Non-stress), **1D** (time 0), and **1E** (Non-stress)). A disulfide-reducing agent, DTT, and an N-glycosylation inhibitor, tunicamycin, are the two most widely used ER stressors, which evoke potent UPR trough disrupting protein folding in the ER in a wide variety of eukaryotic species. **Fig. 1C** also shows that the low splicing levels of *HAC1* mRNA that was induced by low concentrations of DTT or tunicamycin was substantially amplified when cells were cultured under the static condition. This was confirmd by a time-course experiment shown in **Fig. 1D**. However, this difference between the aerobically shaken and static conditions was compressed when cells were strongly ER-stressed by high concentrations of DTT or tunicamycin (**Fig. 1C**). We and others previously reported that the UPR is induced by addition of ethanol into *S. cerevisiae* cultures [7, 8]. **Fig. 1E** demonstrates that the splicing of *HAC1* mRNA that was induced by ethanol was compromised when the cultures were aerobically shaken.

**Figure 2:**
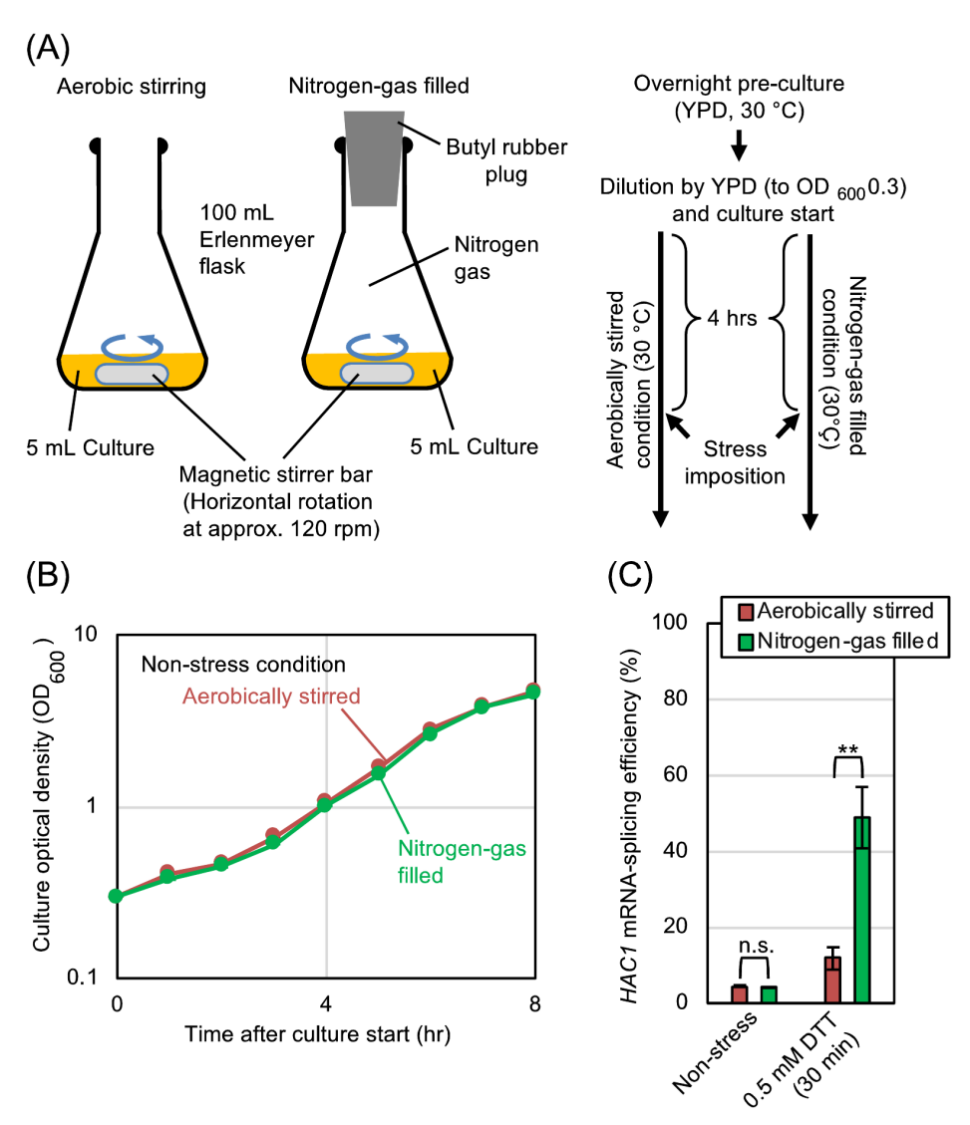
FIGURE 2: Hypoxic treatment boosts *HAC1*-mRNA splicing even in agitated cultures. **(A)** Procedures for culturing and ER-stress induction of *S. cerevisiae* cells under the aerobically stirred condition or under the nitrogen-gas filled condition. DTT was added to the media 4 hr after culture start. **(B)** Optical density of cultures (BY4742 cells) was monitored under non-stress conditions. **(C)** BY4742 cells were cultured and were ER-stressed (or remained non-stressed) as shown in panel A, and were checked for *HAC1*-mRNA splicing. n.s. (not significant): p > 0.05, **: p < 0.01.

We subsequently explored the relationship between the UPR and the aeration status more deeply using DTT as a model ER-stress inducer. In order to ascertain that the difference in the ER-stress status between the static and shaken cultures shown in **Fig. 1** is actually due to their aeration status, we employed another culturing procedure, which is shown in **Fig. 2A**. After being pre-cultured under the aerobically shaken condition, cells were further cultured aerobically along with agitation by a magnetic stirrer. In order to culture cells under a deaerated condition along with agitation, flasks were filled with nitrogen gas during stirring. As shown in **Fig. 2B**, the growth rates of *S. cerevisiae* cells under these two conditions were almost the same. Meanwhile, *HAC1*-mRNA splicing that was induced by dilute DTT was boosted by culturing cells under the nitrogen gas-filled condition (**Fig. 2C**).

In order to further ascertain that the static culturing and the inert-gas fill enhance the *HAC1*-mRNA splicing for the same reason, we performed the experiment shown in Fig. S1. In agreement with the aforementioned observations, the *HAC1*-mRNA splicing in cells treated with 0.5 mM DTT was almost equally boosted by culturing cells under the static condition or the inert gas-filled conditions. Importantly, the static culturing and the inert-gas fill did not seem to act additively to boost the *HAC1*-mRNA splicing. We thus assume that these conditions commonly enhance the *HAC1*-mRNA splicing via deaeration.

**Figure 3:**
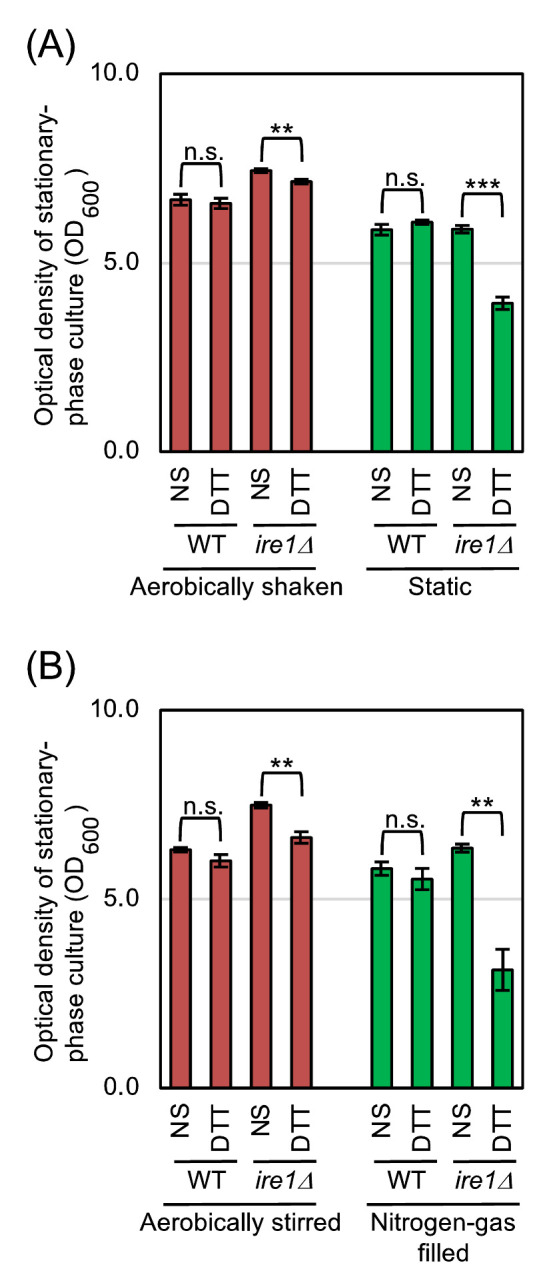
FIGURE 3: Hypoxic treatment aggravates ER stress-induced cellular damage. **(A)** Wild-type BY4742 cells and the congenic *ire1*Δ mutant were cultured as shown in Fig. 1A, and optical density of the cultures was measured 18 hr after culture start. For DTT treatment, we added DTT (0.5 mM final conc.) into media 4 hr after culture start, and further performed the culturing for 14 hr. **(B)** The same strains used in panel A were cultured as shown in Fig. 2A, and optical density of the cultures was measured 18 hr after culture start. For DTT treatment, we added DTT (0.5 mM final conc.) into media 4 hr after culture start, and further performed the culturing for 14 hr. n.s. (not significant): p > 0.05, **: p < 0.01, ***: p < 0.001.

We subsequently investigated whether *S. cerevisiae* cells are more severely damaged by ER stress with low-dose DTT under hypoxic conditions than under aerated conditions. As described in the Introduction section, Ire1 triggers the UPR, which then subdues ER stress. The *IRE1*-gene knockout mutation thus causes hypersensitivity of *S. cerevisiae* cells to ER stress [9]. As shown in **Fig. 3A** and **3B**, we monitored culture density 14 hr after DTT imposition (18 hr after culture start), wherein the cultures had reached the stationary phase. *S. cerevisiae* cells that were weakly ER-stressed by 0.5 mM DTT only poorly grew when carrying the *ire1*Δ mutation and cultured under either the static condition or the nitrogen gas-filled condition.

**Figure 4:**
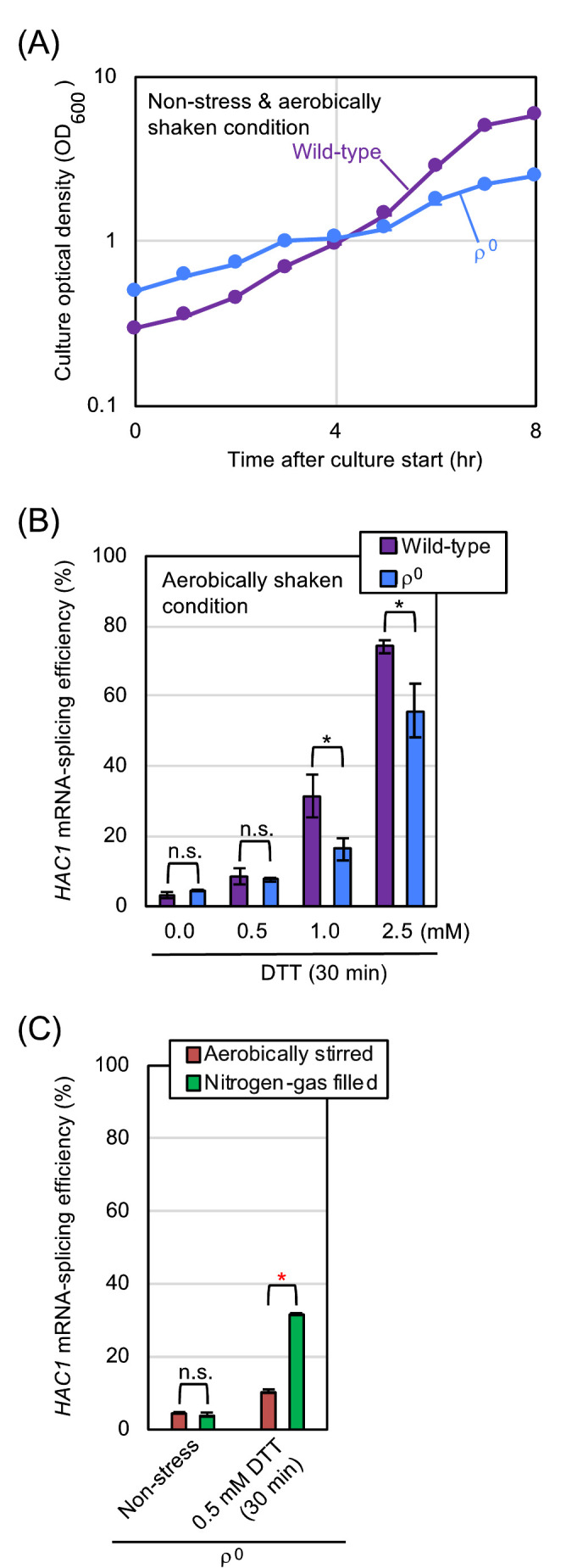
FIGURE 4: Hypoxic treatment boosts *HAC1*-mRNA splicing even in ρ^o^ mutant cells. **(A)** and **(B)** Wild-type BY4742 cells and the congenic ρ^o^ mutant were cultured (or ER-stressed by the indicated concentrations of DTT for 30 min) under the aerobically shaken condition at 30°C in YPD, and were checked for optical culture density and *HAC1*-mRNA splicing. **(C)** The ρ^o^ mutant cells used in panels A and B were cultured and ER-stressed as shown in Fig. 2A, and were checked for *HAC1*-mRNA splicing. n.s. (not significant): p > 0.05, *: p < 0.05, **: p < 0.01.

Our observations presented so far demonstrate that aeration mitigates ER stress in *S. cerevisiae* cells. We then investigated whether mitochondrial respiration, which requires molecular oxygen, is involved in this phenomenon. *S. cerevisiae* ρ^0^ mutant does not carry mitochondrial DNA, and is therefore unable to perform mitochondrial respiration. In the experiments shown in **Fig. 4A** and **4B**, ρ^0^ cells and congenic wild-type cells were cultured under the aerobically shaken condition. Probably because of impairment of mitochondrial functions, ρ^0^ cells grew slower than wild-type cells. Meanwhile, the *HAC1*-mRNA splicing that was induced by DTT was not boosted, but was somewhat compromised by the ρ^0^ mutation. We thus presume that the *HAC1* mRNA-splicing in ρ^0^ cells is lower than that in equally ER-stressed wild-type cells possibly because of the difference in their growth rates. In other words, when compared to that of wild-type cells, the ER-stress status of ρ^0^ cells can be underestimated by checking the *HAC1* mRNA-splicing efficiency.

We next cultured ρ^0^ cells with stirring under the aerated or nitrogen gas-filled condition (**Fig. 4C**). Similar to the case of wild-type cells (**Fig. 2C**), the *HAC1*-mRNA splicing that was induced by low-dose DTT was boosted by culturing ρ^0^ cells under the nitrogen gas-filled condition. These observations indicate that aeration mitigates ER stress, at least partially, via a mechanism(s) independent of mitochondrial respiration. In the experiment shown in Fig. S2, a similar experiment as done in **Fig. 1C** was performed using ρ^0^ cells and tunicamycin. As well as the case of wild-type cells, treatment of ρ^0^ cells with low-dose tunicamycin caused higher-level *HAC1*-mRNA splicing in the static culture than in the aerobically shaken culture.

**Figure 5:**
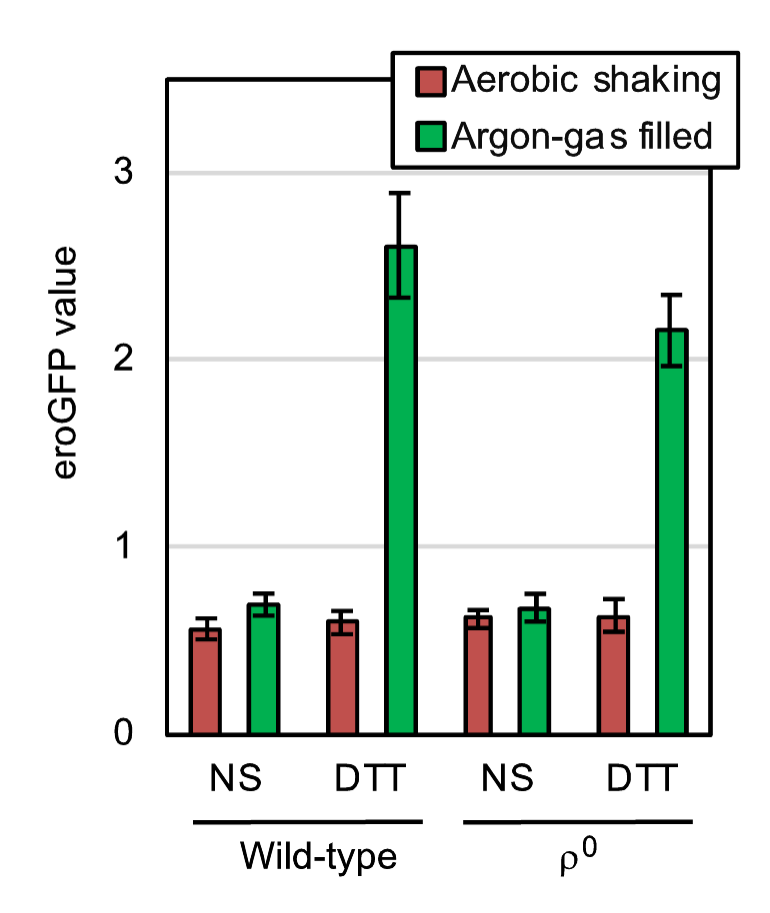
FIGURE 5: Hypoxic treatment potentiates ER stress-induced impairment of the cysteine disulfide bond-formation in the ER of both wild-type and ρ^o^ mutant cells. At 30°C in YPD, wild-type BY4742 cells and the congenic ρ^o^ mutant carrying the pPM28 eroGFP-expression plasmid were aerobically grown, and were stressed by 0.5 mM DTT (or remained non-stressed (NS)) under the aerobic shaking or argon gas-filled condition for 30 min. The cells were then fluorescence-microscopically observed using two different excitation lights for measurement of the eroGFP values, the calculation method of which is described in the Materials and Methods section.

Disulfide-bond formation is an important step in protein folding in the ER, which is thus referred to as oxidative protein folding. As described later, it is highly likely that molecular oxygen is directly involved in this cellular event. In order to determine the cellular ability to form cysteine disulfide bonds in the ER, we used the eroGFP reporter, which is an ER-located GFP variant and changes its fluorescence excitation spectrum dependent on the formation of an intramolecular cysteine disulfide bond [10]. In the experiments shown in **Fig. 5**, *S. cerevisiae* cells expressing the eroGFP reporter were observed under a fluorescence microscope with two different excitation lights. Cells emit bright fluorescence only upon excitation with blue light (high eroGFP ratio) when the disulfide-bond formation in eroGFP is inhibited, whereas oxdatively folded eroGFP is efficiently excited by UV/violet light. For this assay, cells were aerobically cultured in order to allow maturation of the eroGFP fluorophore, and were set into the aerobic shaking condition or the argon gas-filled static condition immediately after the ER-stress onset. The treatment of wild-type ρ^+^ cells with diluted DTT significantly increased the eroGFP value only under the argon gas-filled hypoxic condition (**Fig. 5**). Moreover, a similar result was obtained when cells carried the ρ^0^ mutation (**Fig. 5**). We therefore postulate a mitochondrial respiration-independent role(s) of molecular oxygen to mitigate ER stress and to promote oxidative protein folding. Nevertheless, we do not intend to argue that mitochondrial respiration does not contribute to the mitigation of ER stress by aeration, because, as described later, it is possible that ER stress is mitigated by aeration both dependently and independently of mitochondrial respiration.

**Figure 6:**
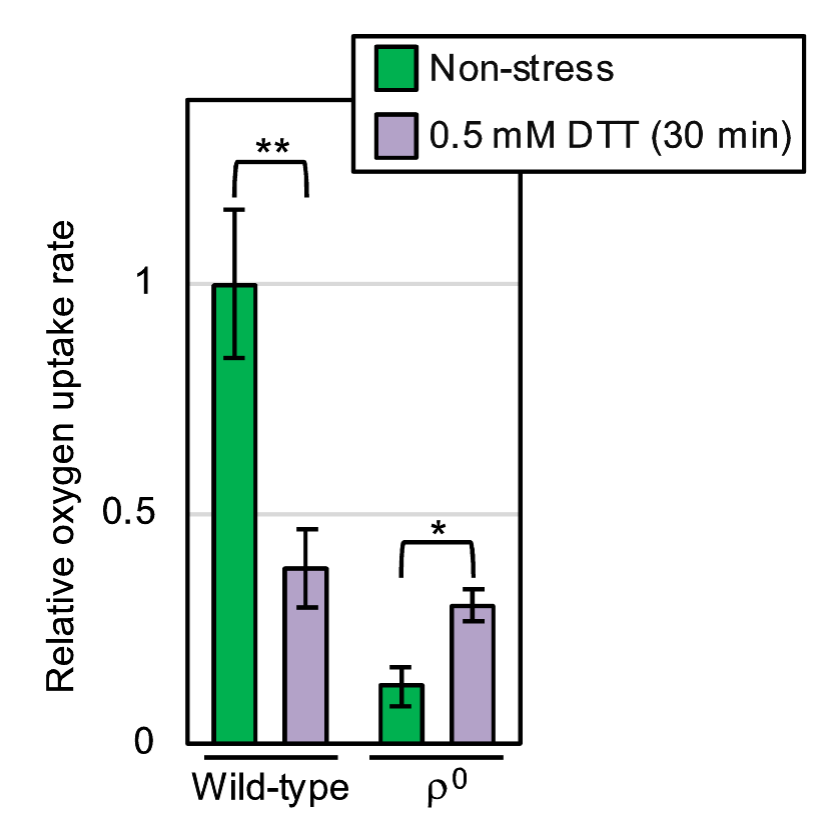
FIGURE 6: Oxygen consumption of wild-type and ρ^0^ yeast cells under non-stress and ER stress conditions. After being aerobically shaken at 30°C, YPD cultures of wild-type BY4742 cells and those of the congenic ρ^o^ mutant were subjected to measurement of the oxygen-consumption rate in the presence or absence of 0.5 mM DTT. The oxygen uptake ratios were the calculated as described in the Materials and Methods sections, and are normalized against that of non-stressed wild-type cells, which is set at 1.0. *: p < 0.05, **: p < 0.01.

We next measured molecular oxygen consumption of yeast cultures using a dissolved oxygen meter. As shown in **Fig. 6**, oxygen consumption of non-stressed yeast cultures was nearly completely abolished by the ρ^0^ mutation (compare the third column to the leftmost column). This observation is consistent with the commonly accepted idea that molecular oxygen is mainly consumed by mitochondrial respiration in eukaryotic cells. According to a previous report by others [11], ER stress stimulates oxygen consumption of yeast cultures. However, in our hands, the oxygen consumption of wild-type ρ^+^ cells was decelerate by DTT exposure (**Fig. 6**; compare the second column to the leftmost column). In contrast, DTT accelerated the oxygen consumption of ρ^0^ cells (**Fig. 6**; compare the rightmost column to the third column).

How do aeration and oxygen consumption lead to the mitigation of ER stress independently of mitochondrial respiration? For ergosterol biogenesis, lipidic intermediates are subjected to multi-step oxidization, in which molecular oxygen is involved, on the ER membrane of *S. cerevisiae* cells [12]. Moreover, membrane lipid-related abnormalities are likely to activate Ire1 directly [6, 13]. One possible scenario hypothesized from these insights is that hypoxia causes ergosterol deficiency, leading to induction or aggravation of ER stress. However, the *HAC1*-mRNA splicing induced by cellular treatment with low-dose DTT under the anaerobic condition was not mitigated, but was slightly enhanced by addition of ergosterol into culturing medium (Fig. S3).

**Figure 7:**
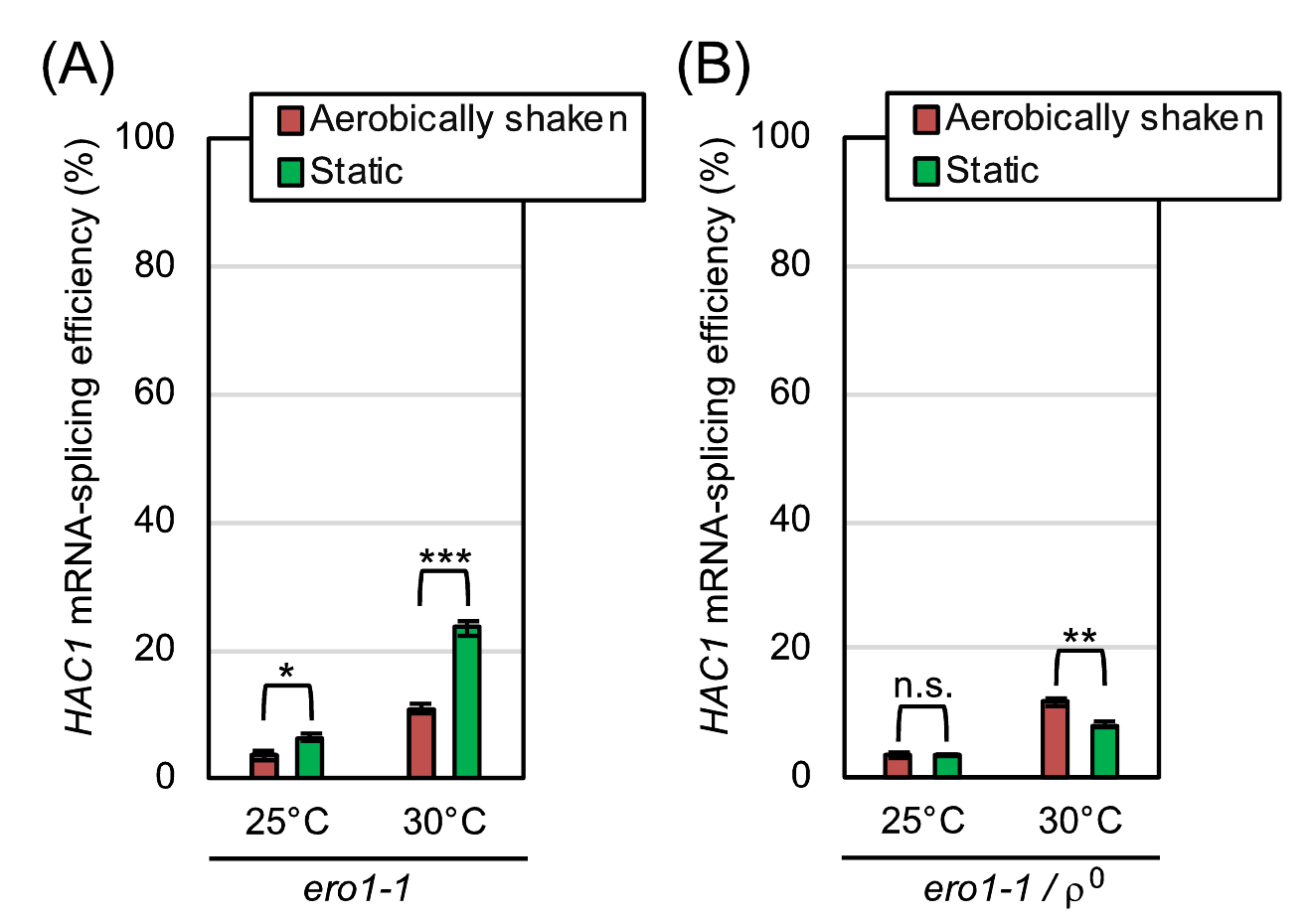
FIGURE 7: ER-stress induced by the *ero1-1* mutation is alleviated by aerobic agitation dependently on mitochondrial respiration. After being pre-cultured at 25°C in YPD, TSA203 cells, which carried the *ero1-1* mutation **(A)**, and the congenic ρ^o^ mutant **(B)** were cultured at 25°C or 30°C under the indicated conditions in YPD for 4 hr, and were checked for *HAC1*-mRNA splicing. n.s. (not significant): p > 0.05, *: p < 0.05, **: p < 0.01, ***: p < 0.001.

According to an *in vitro* investigation using purified Ero1-protein samples [14], molecular oxygen works to directly oxidize Ero1 and to start the ER oxidation cascade. If the mitochondrial respiration-independent mitigation of ER stress by aeration is due to this Ero1-mediated reaction, it may not be observed when Ero1 is deactivated. *ERO1* is an essential gene, and cells carrying the temperature-sensitive *ero1-1* allele suffer from ER stress when cultured at high temperatures even without external stress stimuli [15]. This observation was reproduced in our experiments shown in **Fig. 7**, in which *ero1-1* cells were cultured at the permissive temperature of 25°C or at the semi-permissive temperature of 30°C. As shown in **Fig. 7A**, respiration-capable (ρ^+^) *ero1-1* cells being cultured under the shaking condition at 30°C exhibited a modest *HAC1*-mRNA splicing, which was boosted by culturing cells less aerobically under the static condition. In contrast, the *HAC1*-mRNA splicing was not boosted but was slightly suppressed by the static culturing, when the *ero1-1* cells carried the ρ^0^ mutation (**Fig. 7B**).

As explained more in detail later in the Discussion section, our observations presented so far can be explained by the following scenario. We assume that aeration mitigates ER stress through both the mitochondrial respiration-dependent way and the Ero1-dependent way. Therefore, the *HAC1*-mRNA splicing induced by low-dose DTT or tunicamycin was compromised by aerobic agitation of cultures even when cells carried the ρ^0^ mutation (**Fig. 4C** and S2). On the other hand, we deduce that aeration mitigates ER stress caused by the Ero1 dysfunction strictly in a mitochondria-dependent manner, because this phenomenon was not observed when cells carried the ρ^o^ mutation (**Fig. 7**).

An observation that is seemingly discrepant with this scenario is that the *HAC1*-splicing level in *ero1-1* ρ^0^ cells was somehow lower than that in *ero1-1* cells (ρ^+^) especially under anaerobic conditions (**Fig. 7**; compare panel B to A). We do not think that this observation indicates that *ero1-1* ρ^0^ cells experienced a weaker ER-stress than *ero1-1* cells because, as aforementioned, we may underestimate the ER-stress status of ρ^0^ cells through the *HAC1* mRNA-splicing analysis. In the experiment shown in Fig. S4, *ero1-1* cells and *ero1-1* ρ^0^ cells were anaerobically cultured at 30 °C and further stressed by DTT, which boosted the *HAC1*-mRNA splicing. This observation indicates that, under the conditions employed in **Fig. 7**, the *HAC1*-mRNA splicing was fully induced neither in *ero1-1* cells nor in *ero1-1* ρ^0^ cells.

We next employed a method other than the *HAC1* mRNA-splicing assay to monitor ER-stressing status of yeast cells. BiP is an ER-located molecular chaperone that captures ER-accumulated unfolded proteins, which are frequently aggregated together with BiP in the ER. In the BiP-sedimentation assay, which was developed in our previous studies [6], cells are broken in the presence of the mild detergent Triton X-100, and are fractionated by ultracentrifugation. When cells have been ER-stressed and abundantly carry unfolded proteins in the ER, the pellet fraction apparently contains BiP, which can be detected by the anti-BiP Western-blot analysis. In the experiments shown in Fig. S5, wild-type cells, *ero1-1* cells, and *ero1-1* ρ^0^ cells were cultured at 30 °C and were subjected to the BiP-sedimentation assay. When *ero1-1* cells and *ero1-1* ρ^0^ cells were anaerobically cultured under the static condition, BiP was clearly detected in the pellet fractions, indicating ER-accumulation of unfolded proteins in these cells. Importantly, aerobic agitation abolished the BiP sedimentation in *ero1-1* cells but not in *ero1-1* ρ^0^ cells. We thus assume that the ER-stress status was not largely different between *ero1-1* cells and *ero1-1* ρ^0^ cells under anaerobic conditions, and was improved by aeration only in *ero1-1* cells. This observation is consistent with our idea that the ER-related deficiency caused by the *ero1-1* mutation is alleviated by mitochondrial respiration.

## DISCUSSION

The initial aim of this study was to elucidate the effect of aerobic agitation on the physiology of *S. cerevisiae*. Here we focused on ER stress, and monitored the *HAC1* mRNA-splicing efficiency in static cultures and in aerobically shaken cultures. According to our results shown in **Fig. 1**, static culturing alone did not induce the splicing of *HAC1* mRNA. Meanwhile, the *HAC1*-mRNA splicing that was weakly induced by low-dose DTT, low-dose tunicamycin, or ethanol was boosted when cells were cultured under the static condition (**Fig. 1**). This observation implies a benefit of aerobic agitation for industrial ethanol fermentation. Nevertheless, when cells were subjected to strong ER stress by high concentrations of DTT or tunicamycin, *HAC1* mRNA was highly spliced both under the static condition and the aerobically shaken condition (**Fig. 1C**). We think that, under these situations, ER stress was too strong to be mitigated by aeration. We then continued our study using DTT as the representative ER stressor. In the experiments shown in **Fig. 2**, cells were cultured with agitation either in open flasks or in nitrogen gas-filled flasks. This culturing method confirmed our proposal that the *HAC1*-mRNA splicing that is triggered by weak ER stress is boosted by hypoxic conditions, while hypoxia alone does not induce ER stress in *S. cerevisiae* cells. In accordance with the cell-growth assay shown in **Fig. 3** and S2, deficient aeration aggravates cellular damage induced by ER stress.

The mitochondria are the cellular compartments in which molecular oxygen is highly consumed via respiration. Some previous reports by others have touched on the involvement of mitochondria and mitochondrial respiration in cellular responses against ER stress. According to Haynes *et al.* [16], ER stress induces the production of reactive oxygen species (ROS), in which the mitochondria are involved. In agreement with this finding, Knupp *et al.* [11] proposed that, in *S. cerevisiae*, ER stress increases mitochondrial oxygen consumption, which contributes to cellular survival upon ER stress. It is likely that some intracellular signaling pathways mediate the mitochondrial response to ER stress [17]. In mammalian cells, the ER-mitochondrial spatial connection is increased, leading to enhancement of mitochondrial respiration, in response to ER stress [18]. However, at least under our experimental conditions, DTT severely attenuated the oxygen uptake of wild-type *S. cerevisiae* cells (**Fig. 6**). In agreement with this observation, the mitochondrial gene expression in wild-type *S. cerevisiae* cells was drastically decreased by cellular treatment with DTT or tunicamycin [19]. Contrary to the previous observations by others [11, 16–18], ER stress may damage the mitochondria under certain situations. The reason for this discrepancy should be addressed in future. It should also be noted that, as aforementioned, we assume that mitochondrial respiration is partly responsible for the mitigation of ER stress by aeration. In other words, although possibly damaged by ER stress, the mitochondrial respiration alleviates ER stress.

Meanwhile, here we also show that oxygen deprivation aggravates ER stress not only in wild-type ρ^+^ cells but also in ρ^0^ mutant cells (**Fig. 4**). We therefore propose that, in contrast to the aforementioned previous thoughts [11, 17, 18], ER stress is alleviated by aeration, at least partly, in a manner(s) in which mitochondrial respiration is not involved. Intriguingly, DTT accelerated the oxygen uptake of ρ^0^ mutant cells {**Fig. 6**].

In agreement with these observations, the eroGFP reporter assay shown in **Fig. 5** indicated that aeration mitigates the DTT-induced impairment of ER oxidative protein folding both in wild-type cells and in ρ^0^ cells. Meanwhile, unlike the case of DTT-induced ER stress, mitochondrial respiration seemed to be responsible for the mitigation of *ero1-1*-induced ER stress by aerobic agitation (**Fig. 7**). In other words, mitochondria-independent mitigation of ER stress by aerobic agitation was not observed when Ero1 was deactivated. We therefore postulate that molecular oxygen works for mitigation of ER stress in wild-type *S. cerevisiae* cells through two different ways. First, molecular oxygen is an essential factor for mitochondrial respiration. Second, molecular oxygen is consumed in the ER to initiate the Ero1-mediated oxidation cascade. We assume that this scenario is applicable not only to DTT-induced ER stress but also to other ER-stress stimuli, such as tunicamycin, the primary effects of which are not related to the formation of disulfide bonds in ER client proteins. This is because, according to Merksamer *et al.* [10], ER-stress stimuli collectively damage the protein disulfide-bond formation directly or indirectly.

As aforementioned, the function of molecular oxygen in the ER has been initially proposed through *in vitro* biochemical studies. According to Tu and Weissman [14], molecular oxygen directly contributes to oxidization of Ero1. Our results of this study provide an *in vivo* cell-biological evidence that argues for the physiological importance of the direct involvement of molecular oxygen in the oxidative protein folding in the ER.

*S. cerevisiae* is a facultative anaerobic organism, which grows well under both aerobic and hypoxic conditions in the presence of fermentable carbon sources. In conclusion, here we demonstrate a scene in which aeration is beneficial for *S. cerevisiae* cells even under glucose-rich fermentative conditions. It is widely known that oxygen deprivation causes ER stress and incudes the UPR in mammalian cells [20, 21]. This is frequently observed in cancer cells, which stay under hypoxic conditions and proliferate independently of mitochondrial respiration [5, 20]. We anticipate that, in future, we can address more deeply the mechanism by which hypoxia induces or aggravates ER stress using *S. cerevisiae* as a model organism.

## MATERIALS AND METHODS

### Yeast media

YPD medium contained 1% yeast extract (Bacto), 2% peptone (Bacto), and 2% glucose. YPG medium contained yeast extract (Bacto), 2% peptone (Bacto), and 3% glycerol. For preparation of agar plates, the media were solidified with 2% Bacto agar.

### Yeast strains

Throughout this study, we employed the standard *S. cerevisiae* strain BY4742 (*MATα his3*Δ*1 leu2*Δ*0 lys2*Δ*0 ura3*Δ*0*) [22] as the wild-type strain. The *ire1*Δ (*ire1::kanMX4*) derivative of BY4742 was obtained from EUROSCARF (Y11907; http://www.euroscarf.de/). BY4741 (*MAT****α*** *his3*Δ*1 leu2*Δ*0 met15*Δ*0 ura3*Δ*0*) is congenic with BY4742, and its *ero1-1* derivative (TSA203) was also obtained from EUROSCARF. Yeast strains were grown at 30°C (or at 25°C for TSA203) in YPD medium containing 10 µg/mL ethidium bromide (EtBr) for approximately 15 generations, and the progenies that could not grow on YPG agar plates were employed as ρ^o^ mutant strains.

### Yeast plasmid and transformation

Yeast plasmid pPM28 (the *URA3* selectable marker) [10] carries the eroGFP gene, the expression of which is controlled by the constitutive *TDH3* promoter, and was obtained from Addgene (https://www.addgene.org/). The low-copy *IRE1* plasmid pRS313-IRE1 and the empty vector pRS313 were described previously [23, 24]. Yeast transformation was performed as described in Kaiser *et al.* [25].

### Yeast culturing conditions

Unless otherwise noted, for pre-culturing, *S. cerevisiae* strains were inoculated into YPD medium, and were incubated overnight under the aerobically shaken condition at 30°C. The cultures were subsequently diluted with YPD to OD_600_ 0.30 (or to OD_600_ 0.50 for the ρ^o^ mutants), and were further incubated at 30°C for 4 hr before stress imposition and harvesting. For the aerobically shaken condition, 50 mL polypropylene conical tubes (Nichiryo) containing 5 mL of the cultures were loosely capped, were kept in a slanting manner, and were shaken in the recipro shaker Taitech Personal Lt-10F (40 mm stroke, 160 rpm). For the static condition, 15 mL polypropylene conical tubes (Nichiryo) containing 5 mL of the cultures were tightly capped and were vertically stood without shaking. Alternatively, 5 mL of the cultures were stirred in 100 mL Erlenmeyer flasks (Iwaki) with magnetic stir bars at approximately 120 rpm. In order to deaerate the stirring cultures, the Erlenmeyer flasks were filled with nitrogen gas, and were tightly plugged with butyl rubber plugs. The *ero1-1* strains were pre-cultured at 25°C.

Culture density was monitored using the spectrophotometer Smartspec 3000 (BioRad).

### Stress imposition

Dithiothreitol (DTT; Nakarai Tesque; 1 M stock solution in water), tunicamycin (Sigma-Aldrich; 2 mg/mL stock solution in dimethylsulfoxide) and ethanol were added into the medium 4 hr after culture start, and the culturing was continued without changing the aeration status. When ER stress-inducing agents were added into the static cultures, the conical tubes were immediately and gently inverted five times for mixing.

### RNA analysis

Total RNA samples were extracted from *S. cerevisiae* cells using the hot-phenol method, and were subjected to reverse transcription (RT)-PCR analysis using the poly(dT) RT primer and the *HAC1*-specific PCR primers [6, 25]. Because the *HAC1* specific PCR primers interposes the *HAC1* intron sequence, the unspliced and spliced variants of *HAC1* mRNA yielded different-sized RT-PCR products, which were separated by electrophoresis on 2% agarose gel. Fluorescent images of the EtBr-stained gels were analyzed by the Image J image-processing software (https://imagej.nih.gov/ij/) for calculating the “*HAC1*-mRNA splicing efficiency” through the following formula:

*HAC1* mRNA-splicing efficiency = 100X(Band intensity of the spliced species)/{(Band intensity of the spliced species)+(Band intensity of the unspliced species)}.

### Fluorescence microscopy

Cells carrying the eroGFP-expression plasmid pPM28 were grown in YPD medium at 30°C under the aerobic shaking condition, and DTT (0.5 mM final) was added (or not added) into cultures. Subsequently, the shaking incubation was additionally continued at 30°C for 30 min, or the cultures were set into argon gas-filled glass-bottom dishes and statically incubated at 30°C for 30 min. The eroGFP-fluorescent images of *S. cerevisiae* cells were then observed under the laser scanning microscopy SP8 FALCON (Leica) with the 63x/1.40 HC PL APO CS2 objective lens. For excitation, the 405 nm diode laser (UV/violet-light excitation, 25% output) and the 487 nm white-light laser (blue-light excitation, 70% output) were employed. For detection, the hybrid detector (gating 492-527 nm) was employed. The pinhole size was 1.00 AU. Fluorescence intensities of a cell illuminated by these two lights were respectively quantified using the Image J software (50 cells were analyzed from each sample), and were used to calculate the “eroGFP value” through the following formula:

eroGPF value = (Fluorescence intensity with blue-light excitation)/(Fluorescence intensity with UV/violet-light excitation)

### Oxygen consumption assay

YPD cultures were aerobically agitated until their OD_600_ reached approximately 2.0. Then, 10 mL of the cultures were transferred to 50 mL polypropylene conical tubes, which were subsequently filled with argon gas and were set in a 30 °C water bath. Dissolved oxygen concentration of the cultures was monitored for 3 min by inserting a dissolved oxygen electrode (Dissolved Oxygen Meter AR8210 (Smart Sensor)) into the conical tubes. For DTT exposure, DTT (0.5 mM final) was added into the cultures 30-min before the measurement. The “oxygen uptake rate” value is normalized against the culture optical density (OD_600_).

### BiP sedimentation assay

Cells equivalent to 1.0 OD_600_ were broken by bead-beating in 100 µL of the lysis buffer containing 50 mM Tris-Cl (pH 7.9), 5 mM ethylenediaminetetraacetic acid, 1% Triton X-100 and protease inhibitors (2 mM phenylmethylsulfonyl fluoride, 100 μg/ml leupeptin, 100 μg/ml aprotinin, 20 μg/ml pepstatin A and Calbiochem Protease Inhibitor cock-tail Set III (100× dilution)). The crude cell lysates were clarified by centrifugation at 8,000×g for 10 min, and were used as the total cell lysate samples, which were further centrifuged at 87,000×g for 30 min (ultra-centrifugation). The total cell lysate samples and the ultra-centrifugation pellet samples were run on 8% SDS-PAGE, which was followed by anti-BiP Western blotting [26].

### Statistics

The culture optical density, the *HAC1* mRNA-splicing efficiency, and the oxygen uptake rate were determined from triplicate cultures, and are subjected to calculation of averages and standard deviations, which are presented in the figures. In order to obtain *p* values, we performed two-tail unpaired t-Test using Microsoft Excel.

## SUPPLEMENTAL MATERIAL

Click here for supplemental data file.

All supplemental data for this article are available online at http://www.microbialcell.com/researcharticles/2021a-phuong-microbial-cell/.

## Abbreviations:

ER: – endoplasmic reticulum,
PDI: – protein disulfide isomerase,
UPR: – unfolded protein response.
