# Supplementary material for: Aeration mitigates endoplasmic reticulum stress in Saccharomyces cerevisiae even without mitochondrial respiration
Source: Microb Cell. 2021 Mar 31;8(4):77–86. doi: 10.15698/mic2021.04.746 (PMC8010904; doi:10.15698/mic2021.04.746)
Supplement: Supplementary file 1 [file mic-08-077-s01.pdf]

Figure S1

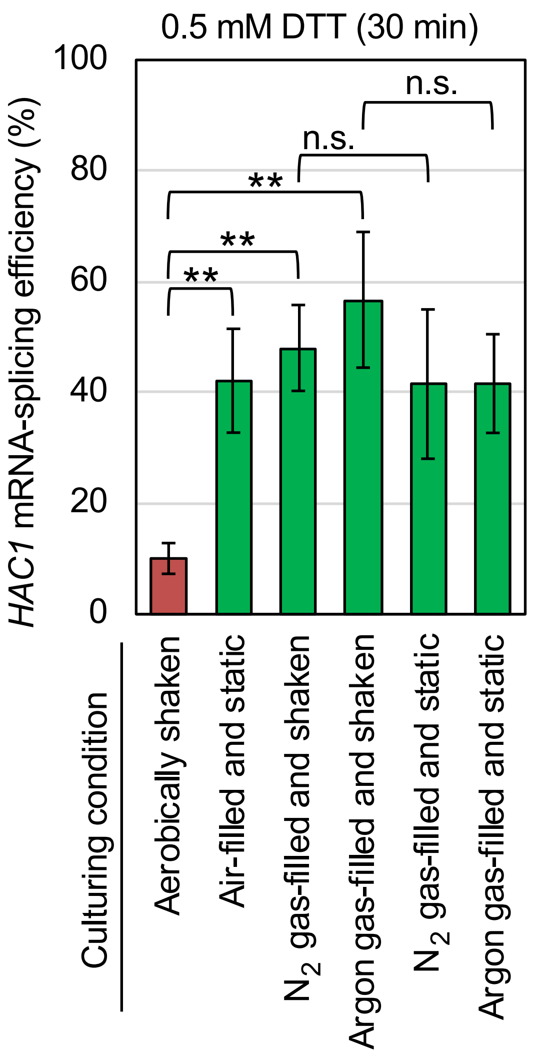

Figure S1 Hypoxic conditions commonly and non-additively boost DTT-induced ER stress.
YPD cultures of wild-type BY4742 cells (5 mL) were incubated at 30 ° C statically or with shaking (160 rpm) in 50 mL conical tubes filled with the indicated gases. DTT (0.5 mM final conc.) was added into all cultures 30-min before harvest.

Figure S2

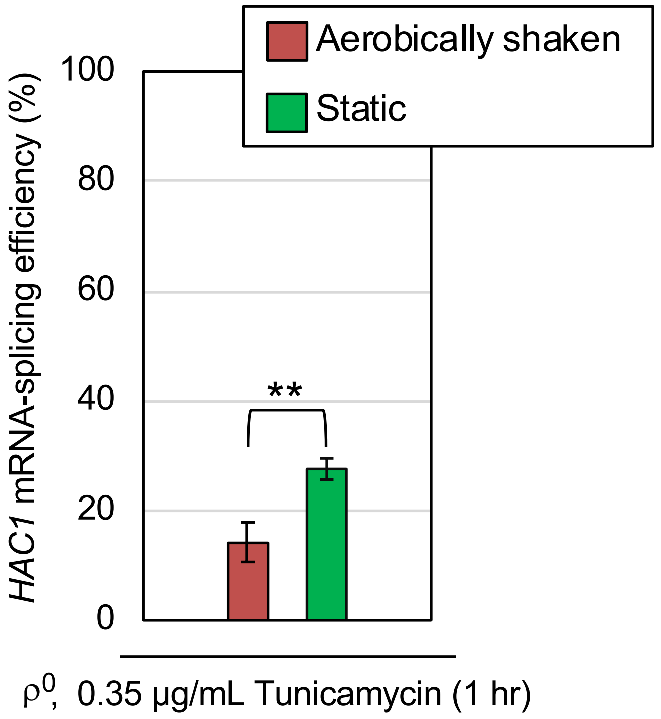

Figure S2 Aerobic agitation mitigates tunicamycin-induced ER stress even when cells carry the ρ^0 mutation.
The ρ^o mutant of BY4742 was cultured at 30 ° C in YPD under the static or aerobically shaking condition. Tunicamycin (0.35 μg/mL) was added into all cultures 1-hr before harvest.

Figure S3

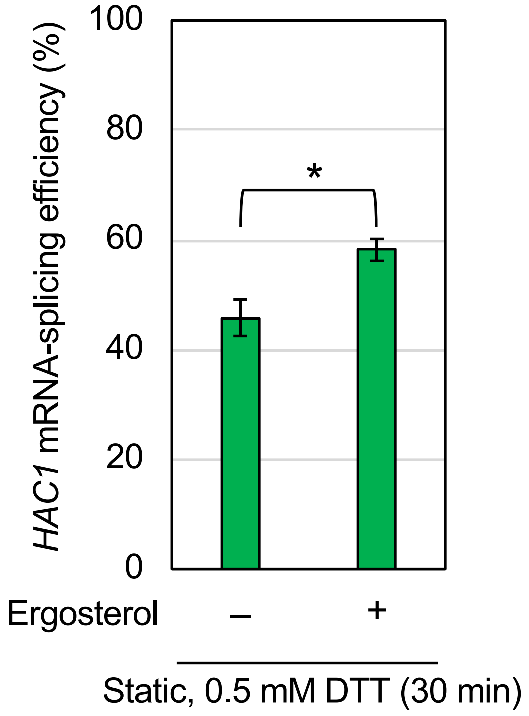

Figure S3 Ergosterol does not mitigate ER stress boosted by deaeration. Wild-type BY4742 cells were cultured at 30 ° C in YPD under the static condition. DTT (0.5 mM final conc.) was added into all cultures 30-min before harvest. For the "+Ergosterol" samples, YPD was supplemented with ergosterol (10 μg/mL final conc.). *: $p < 0.05$.

Figure S4

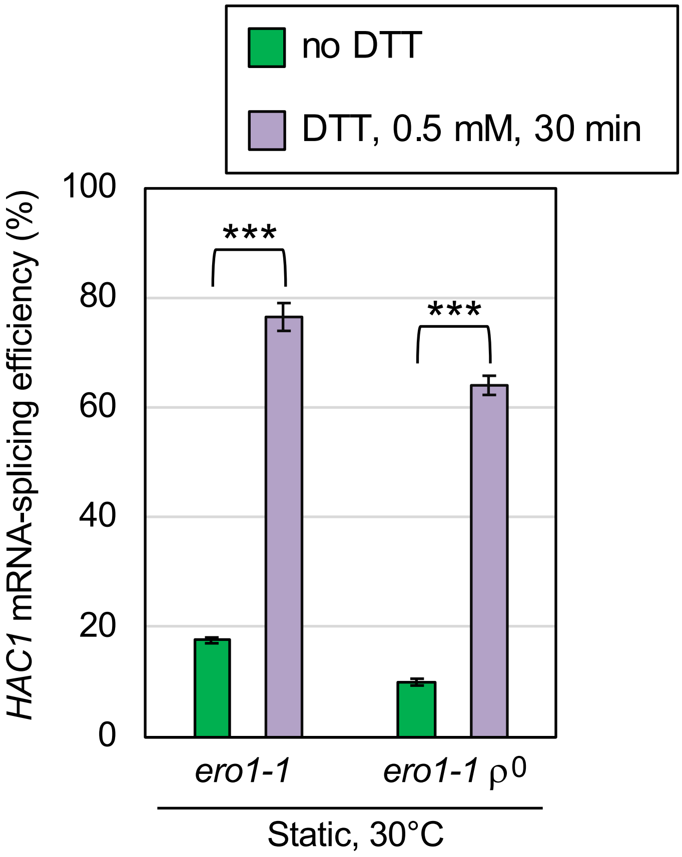

Figure S4 DTT boosts ER stress induced by the *ero1-1* mutation.
As done in the experiments shown in Fig. 7, TSA203 cells (*ero1-1*) and the congenic ρ° mutant cells were cultured at 30 ° C in YPD under the static condition. For the "+DTT" samples, DTT (0.5 mM final conc.) was added into cultures 30-min before harvest. n.s. (not significant): $p > 0.05$, ***: $p < 0.001$.

Figure S5

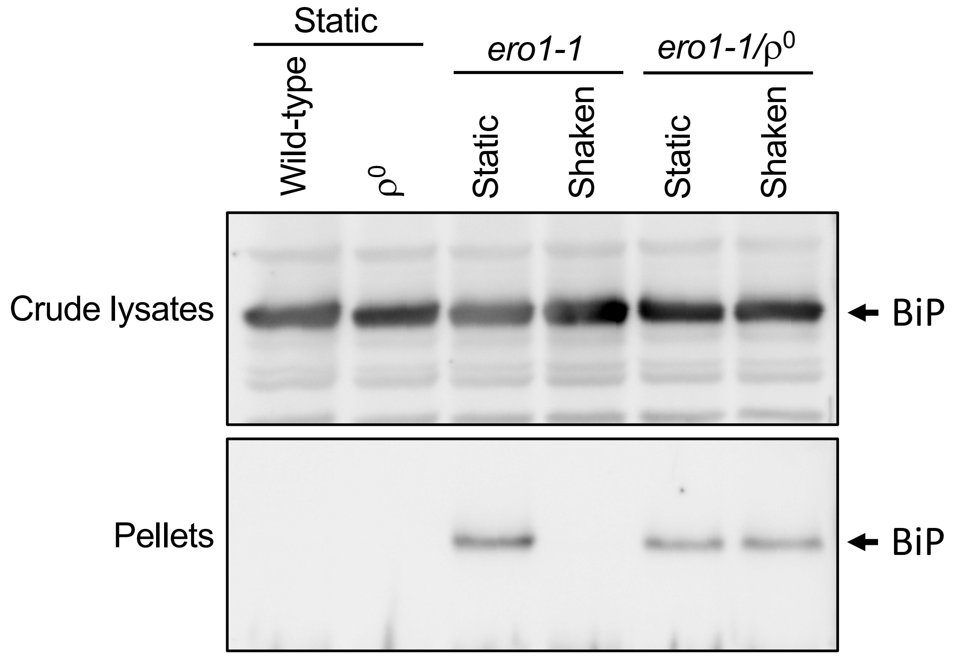

Figure S5 Aeration mitigates *ero1-1*-induced BiP sedimentation in ρ^+ cells bot not in ρ^0 mutant cells.

Wild-type BY4742 cells, TSA203 cells (*ero1-1*), and their congenic ρ^o mutant cells were grown in YPD at 30 ° C under the indicated conditions for 5 hr. Total cell lysates were then obtained and fractionated by ultra-centrifugation for the BiP sedimentation assay. The figure represents anti-BiP Western blot images of the total cell lysates (equivalent to 0.025 OD_{600} cells) and the pellet samples (equivalent to 0.25 OD_{600} cells).
